# Elevated In Vitro Kinase Activity in Peripheral Blood Mononuclear Cells of Leucine‐Rich Repeat Kinase 2 G2019S Carriers: A Novel Enzyme‐Linked Immunosorbent Assay–Based Method

**DOI:** 10.1002/mds.28175

**Published:** 2020-07-11

**Authors:** Katerina Melachroinou, Min Suk Kang, Christopher Liong, Sushma Narayan, Najah Levers, Neal Joshi, Katie Kopil, Samantha J. Hutten, Marco A.S. Baptista, Shalini Padmanabhan, Un Jung Kang, Leonidas Stefanis, Roy N. Alcalay, Hardy J. Rideout

**Affiliations:** ^1^ Division of Basic Neurosciences Biomedical Research Foundation of the Academy of Athens Athens Greece; ^2^ Department of Neurology Columbia University New York New York USA; ^3^ The Michael J. Fox Foundation for Parkinson's Research New York New York USA; ^4^ Department of Neurology NYU Langone Health New York New York USA; ^5^ Department of Neurology University of Athens Medical School Athens Greece

**Keywords:** ELISA, kinase, LRRK2, Parkinson's disease, PBMC

## Abstract

**Background:**

Leucine‐rich repeat kinase 2 kinase inhibitors are being vigorously pursued as potential therapeutic options; however, there is a critical need for sensitive and quantitative assays of leucine‐rich repeat kinase 2 function and target engagement.

**Objectives:**

Our objective was to compare collection and storage protocols for peripheral blood mononuclear cells, and to determine the optimal conditions for downstream analyses of leucine‐rich repeat kinase 2 in PD cohorts.

**Methods:**

Here, we describe enzyme‐linked immunosorbent assay–based assays capable of detecting multiple aspects of leucine‐rich repeat kinase 2 function at endogenous levels in human tissues.

**Results:**

In peripheral blood mononuclear cells from both healthy and affected carriers of the G2019S mutation in leucine‐rich repeat kinase 2, we report, for the first time, significantly elevated in vitro kinase activity, while detecting a significant increase in pS935/leucine‐rich repeat kinase 2 in idiopathic PD patients.

**Conclusions:**

Quantitative assays such as these described here could potentially uncover specific markers of leucine‐rich repeat kinase 2 function that are predictive of disease progression, aid in patient stratification, and be a critical component of upcoming clinical trials. © 2020 The Authors. *Movement Disorders* published by Wiley Periodicals LLC on behalf of International Parkinson and Movement Disorder Society.

Mutations in the leucine‐rich repeat kinase 2 (*LRRK2*) gene are a common genetic cause of both familial and sporadic Parkinson's disease (PD). Recent work has demonstrated increased LRRK2 kinase activity in experimental models, and postmortem tissue, of idiopathic PD (iPD^1^, ^2^). Quantitative assays that reliably measure the activity and phosphorylation of LRRK2 are critically needed to predict and monitor progression of disease, and measure efficacy of kinase inhibitors in clinical trials. Delbroek and colleagues^3^ described an enzyme‐linked immunosorbent assay (ELISA) that detected total and pSer935‐LRRK2 in peripheral blood mononuclear cells (PBMCs); however, this assay was not assessed in clinically relevant PD cohorts. By western immunoblotting, phosphorylation of LRRK2 at Ser910 and Ser935 in PBMCs from iPD does not appear to be different in comparison to controls.^4^ LRRK2 is present in exosomes purified from urine,^5^ as well as cerebrospinal fluid,^6^ and significantly elevated pSer1292‐LRRK2 is detected in affected carriers of the G2019S mutation.^7^ Other assays have been described that assess the kinase inhibitor‐dependent phosphorylation of the LRRK2 substrates, Rabs 10 and 12, by western immunoblotting and mass spectrometry–based techniques.^8^, ^9^, ^10^

The aim of this study was to identify the optimal PBMC collection and storage protocols for the assessment of LRRK2 in multiple PD cohorts. We measured total and phosphorylated LRRK2 and its in vitro kinase activity. We found that whereas pSer935‐LRRK2 is not significantly altered by the G2019S mutation, despite trending lower compared to controls, the in vitro phosphorylation of a peptide substrate is significantly elevated; while conversely, in iPD PBMCs, in vitro activity is unchanged and pS935‐LRRK2 levels are increased.

## Materials and Methods

### Study Participants

Whole blood and urine were collected from study participants seen at the Department of Neurology, Columbia University Irving Medical Center (New York, NY).^11^ Demographics and clinical characteristics of the cohort are presented in Supporting Information Table S1.

### PBMC Isolation and ELISA Development

A schematic of the PBMC isolation protocols appears in Supporting Information Figure S1, and complete details of the ELISA development are described in the Supporting Information. For the novel sandwich LRRK2 ELISA, rabbit monoclonal anti‐LRRK2 (clone c41‐2; Abcam, Cambridge, MA) was used as the capture antibody and either mouse monoclonal anti‐LRRK2 (clone N241A; NeuroMab, Davis, CA) or anti‐pS935 (UDD2; Abcam) as the detector antibodies. For assessment of LRRK2 kinase activity, we used a variation of the assay recently described.^12^

### Statistical Analyses

Descriptive statistics were used to compare the four clinical groups (*LRRK2* G2019S carriers and noncarriers with and without PD). We used chi‐square for dichotomous variables and analysis of variance (ANOVA) for continuous variables. Statistical evaluation of in vitro kinase activity, pS935‐LRRK2 levels, as well as total LRRK2 levels was carried out as indicated using GraphPad Prism software (version 7; GraphPad Software Inc., La Jolla, CA, USA). Groups were compared by unpaired two‐tailed *t* test, Mann‐Whitney U test, or one‐way ANOVA, as indicated, with the number of replicates indicated in the figure legend. To determine whether any clinical features correlate with changes in LRRK2, Spearman's correlation was performed. Significance level was set at *P* < 0.05.

## Results

### Assay Validation

For the complete validation of this ELISA and in vitro kinase assay, please see the accompanying Supporting Information. We utilized recombinant human full‐length LRRK2 (rhLRRK2; Thermo Fisher Scientific, Waltham, MA). Chemiluminescence values were plotted against concentrations of rhLRRK2 and fitted to a regression curve (Supporting Information Fig. S2A), with a lower limit of detection (LLOD) estimated to be ~50 pg/mL.

### Assessment of LRRK2 Intrinsic Kinase Activity in Human PBMCs

**FIG. 1.**
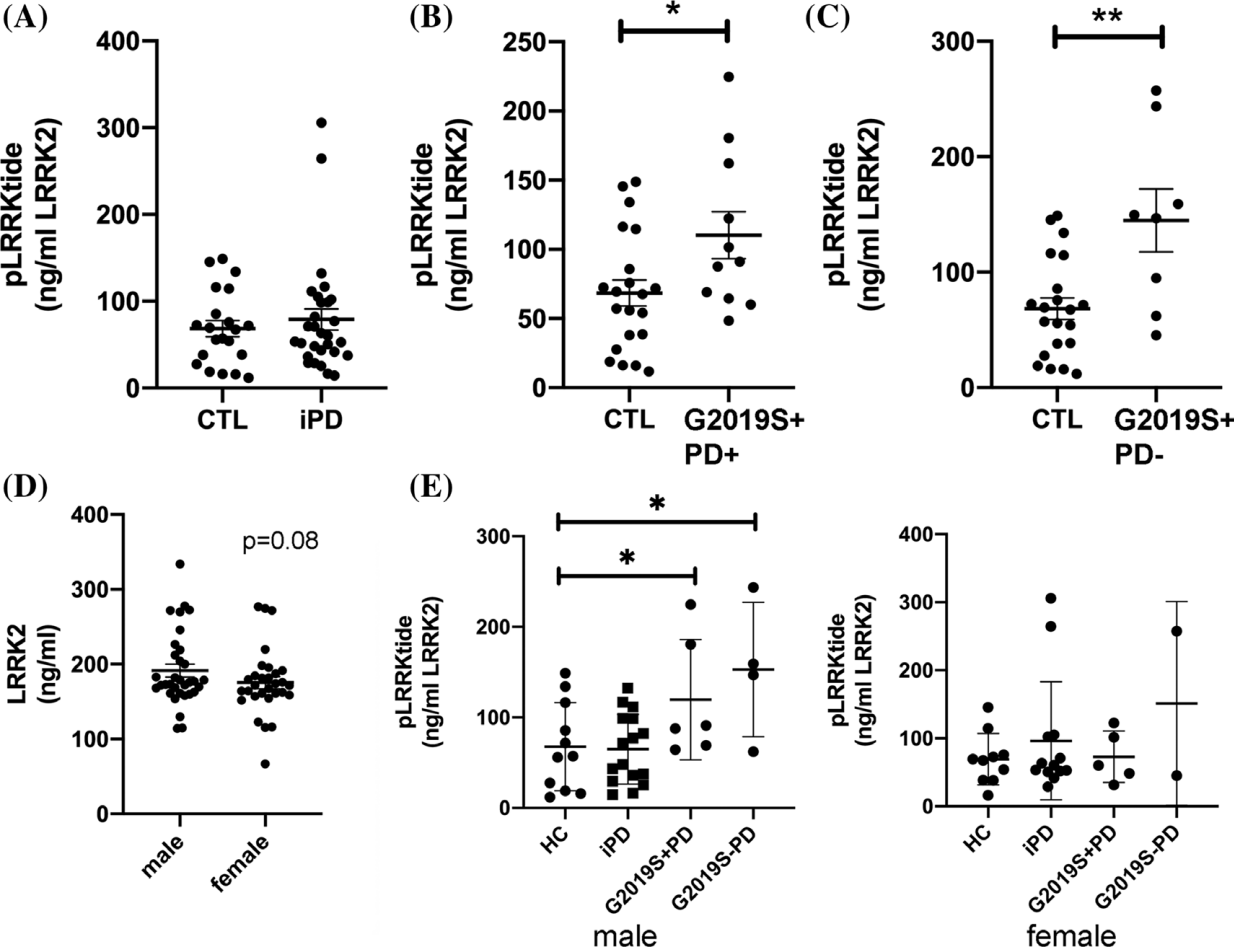
LRRK2 in vitro kinase activity in PBMC extracts is increased in carriers of the G2019S mutation. We measured in vitro kinase activity using a model peptide substrate in an in‐well kinase reaction. Phosphorylation of the peptide was normalized to the total amount of LRRK2 in each well. We could find no significant change in the intrinsic kinase activity of LRRK2 in PBMCs of iPD patients (**A**); however, phosphorylation was significantly increased in affected (**B**) and healthy (**C**) carriers of the G2019S‐LRRK2 mutation (middle and right panels). Mann‐Whitney U test, *P < 0.05; **P < 0.01. (**D**) LRRK2 expression and in vitro activity were compared in PBMCs from males and females. In samples from healthy control and iPD patients, we found a trend for higher levels of expression in PBMCs from males compared to females; Mann‐Whitney U, *P* = 0.08 (Ctl). Interestingly, the increased in vitro kinase activity detected in PBMCs from carriers of the G2019S‐LRRK2 mutation, when analyzed separately by sex, was only evident in PBMCs from male subjects (**E**). The activity of LRRK2 from PBMCs of female G2019S carriers is not different from healthy controls. *P < 0.05. CTL, control; HC, healthy controls; pLRRKtide, phosphorylation of the LRRKtide substrate.

Mean viability of isolated PBMCs did not differ among the protocols (Protocol A: 85.8% ± 0.64; Protocol B: 87.2% ± 0.6; Protocol C: 89.1% ± 0.43). Total expression of LRRK2 was not affected by either disease or LRRK2 mutation status (Supporting Information Fig. S4A). In all isolation methods, we found no significant difference in the in vitro activity of LRRK2 purified from PBMCs from iPD patients in comparison to control subjects (Protocol B; Fig. 1A), suggesting that LRRK2 in these cells does not undergo any modifications in the context of PD that alter its intrinsic kinase activity. In contrast, however, LRRK2 purified from *LRRK2*‐*G2019S* carriers exhibited a significantly increased intrinsic activity (“B” sample; Fig. 1B,C). This was evident in both manifesting as well as nonmanifesting G20129S carriers and reflects the consequences of a localized conformational change in the LRRK2 kinase domain *DYG* motif, predicted to stabilize the activation loop.^13^, ^14^

Not surprisingly, the mean age of healthy carriers of *G2019S‐LRRK2* was significantly less compared to carriers with PD (Supporting Information Fig. S4B). Whereas there was no correlation between age and LRRK2 expression in either *G2019S‐LRRK2* carrier group (Supporting Information Fig. S4D), LRRK2 activity was strongly negatively correlated with age only in the healthy carriers (Supporting Information Fig. S4C). These findings raise the possibility that transient changes in LRRK2 activation may be occurring in such immune cells before phenoconversion in mutation carriers. There was a strong, but not statistically significant, trend for increased LRRK2 expression in PBMCs from males across all groups (Fig. 1D). Moreover, the increased intrinsic activity in LRRK2 isolated from both *G2019S‐LRRK2* groups was only evident in PBMCs from males (Fig. 1E). The reason for these differences is unclear; however, much more work is clearly needed to understand the regulation of LRRK2 and its activity.

### Phosphorylation of LRRK2 at Ser935

**FIG. 2.**
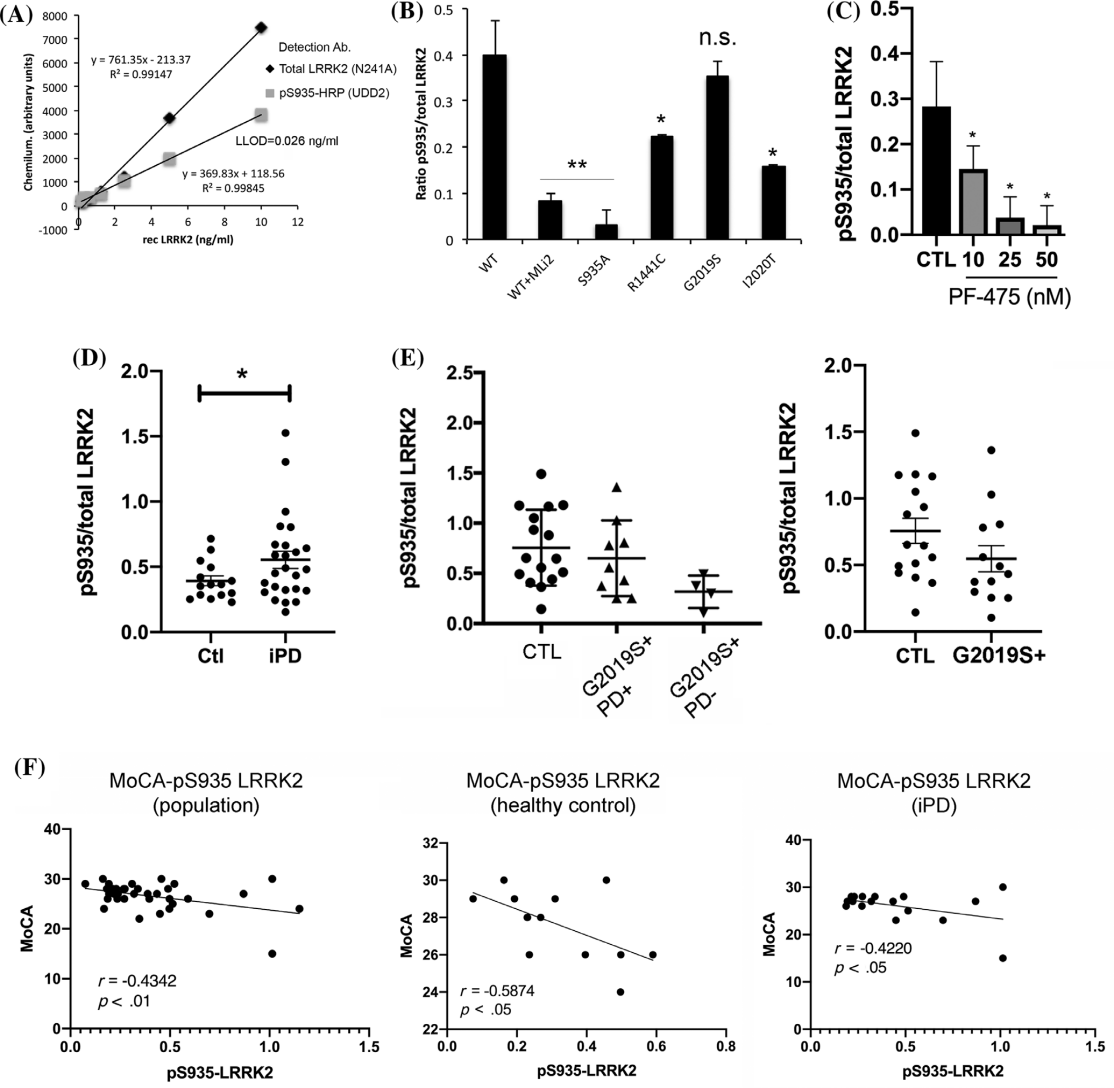
pS935‐LRRK2 ELISA in cell lines and human PBMCs. We assessed phosphorylation of LRRK2 at the Ser935 residue by ELISA. For each sample, the signal corresponding to pS935‐LRRK2 was normalized to its corresponding value of total LRRK2 (in ng/mL). (**A**) Increasing amounts of recombinant human full‐length LRRK2 was bound to parallel ELISA plates using the c41‐2 clone for capture and detected using total (N241A; for normalization) or phosphorylated (pS935‐LRRK2, UDD2) antibodies. (**B**) Extracts of HEK293T cells overexpressing WT (± treatment with MLi‐2; 100 nM) or S935A‐LRRK2 were subjected to ELISA using the anti‐pS935‐LRRK2 antibody either as a capture or detector antibody. In both cases, the signal was significantly lost for S935A‐LRRK2 or following dephosphorylation with the MLi‐2 kinase inhibitor. (**C**) Freshly isolated PBMCs from healthy volunteers were treated with increasing concentrations of the kinase inhibitor, PF‐475, for 1 hour and subjected to pS935‐LRRK2 ELISA. We found a significant dose‐dependent loss of phosphorylated LRRK2. ANOVA and Tukey's post‐hoc comparisons, *P < 0.05; **P < 0.01. We found that pS935‐LRRK2 levels in PBMCs of patients with iPD were significantly higher compared to healthy controls (**D**); *Mann‐Whitney U, *P* < 0.05. In contrast, in PBMC extracts from carriers of the G2019S mutation, with and without PD, there was a nonsignificant trend toward lower levels of pS935‐LRRK2 compared to healthy controls (**E**). Values of all G2019S carriers were pooled for comparison against healthy controls (E, right plot). (**F**) We compared levels of pS935‐LRRK2 and various clinical parameters. In iPD patients, performance on the cognitive function test, MoCA, was negatively correlated with pS935‐LRRK2 levels in iPD patients (F, right panel) and healthy controls (F, center panel). Ab, antibody; CTL, control; HRP, horseradish peroxidase; n.s., not significant; rec LRRK2, recombinant human full‐length LRRK2.

We additionally developed a novel ELISA for the measurement of pS935‐LRRK2, with some validation steps shown in Figure 2A,B. We measured pS935‐LRRK2 levels following treatment of healthy donor PBMCs with the selective inhibitor, PF‐475, and found a dose‐dependent decrease in normalized pS935‐LRRK2 levels following 1‐hour treatment (Fig. 2C). In PBMC extracts from *LRRK2* G2019S carriers, we could find no significant change in normalized pS935‐LRRK2 (Fig. 2E); however, there is an insignificant trend toward lower levels in all *LRRK2* G2019S mutation carriers (Fig. 2E, right panel). Conversely, in PBMCs from iPD patients; we detected a significant increase in pS935‐LRRK2 in comparison to healthy controls (Fig. 2D).

We could find no correlation between pS935‐LRRK2 and UPDRS‐III score in PBMCs from iPD patients (not shown); however, we could detect a significant negative correlation between pS935‐LRRK2 and the Montreal Cognitive Assessment (MoCA) score (Fig. 2F). This correlation was evident not only in PBMCs from iPD patients (Fig. 2F, right panel), but also in PBMCs from healthy control subjects (center panel). This finding is consistent with that of the West group, which reported an association between poorer cognitive function and another measure of LRRK2, increased pSer1292‐LRRK2.^7^ Although the link between increased pS935 levels and increased pS1292 levels remains unclear, these findings indicate that changes in the activation state of LRRK2, pS1292‐LRRK2 in urinary exosomes and pS935‐LRRK2 in PBMCs, may be used to predict cognitive decline.

## Discussion

Using several ELISA‐based assays to the assessment of LRRK2 status in PBMCs from a clinical cohort, we report several key findings. First, we did not detect any differences in total LRRK2 expression levels among the various sample groups, independent of PD or *LRRK2* G2019S mutation status. Second, we found that in vitro kinase activity of LRRK2 from *LRRK2* G2019S mutation carriers was significantly elevated in comparison to noncarriers. Third, we detected a significant elevation in pS935‐LRRK2 in PBMCs from iPD in comparison to controls. Conversely, although we failed to detect significant differences in pS935‐LRRK2 in the *LRRK2*‐*G2019S* population, we did find a trend toward lower pS935‐LRRK2 levels in *LRRK2*‐*G2019S* carriers.

A recent report assessed changes in pS935‐LRRK2 in PBMCs from the same cohort using a proprietary ELISA, based on the Simoa platform.^15^ This report showed a statistically significant decrease in pS935‐LRRK2 in G2019S‐positive PD patients compared to iPD. One possibility for the difference between this report and our current study could be related to the different capture antibodies used in the two studies (rabbit monoclonal, clone c41‐2 here, vs. mouse monoclonal clone N241A in the Simoa assay). It is possible that distinct species of LRRK2 were purified, displaying altered phosphorylation profiles between the two assays. It should be noted that the capture antibody used in the Simoa assay (clone N241A) was the same antibody we have used as the detector in the total LRRK2 ELISA, which, as we have noted in the Supporting Information, returned below threshold signals in several samples where we were able to detect robust in vitro kinase activity. Taken together, given that there was not a consistent link between pS935‐LRRK2 and disease status (increased levels in iPD, decreased levels in G2019S‐PD patients), it appears that the greatest potential use for this specific outcome measure is to assess target engagement following treatment with LRRK2 inhibitors.

Phosphorylation of the LRRKtide substrate is significantly elevated, even with notable variability in the activity values, in carriers of the *LRRK2* G2019S mutation, irrespective of disease status. Conversely, we did not detect a significant change in the intrinsic kinase activity of LRRK2 from iPD subjects. Therefore, in these samples, the phosphorylation of the LRRKtide peptide is associated with mutation status, but not with PD status, at least in PBMCs. In dopaminergic neurons in the iPD postmortem midbrain,^1^ as well as LRRK2 from urinary exosomes,^2^ pSer1292‐LRRK2 levels are elevated. Given that this is an autophosphorylation event, it is indicative of increased *cellular* LRRK2 kinase activity, but does not necessarily reflect changes to the *intrinsic* kinase function of the isolated protein. This is an important distinction to make in the interpretation of these data; that the lack of an increase in the activity of LRRK2 purified from iPD PBMCs is not incompatible with previous reports of increased activation LRRK2 in iPD samples. Using a similar assay, with overexpressed mutant LRRK2, we have previously reported that phosphorylation of LRRKtide is not increased in isolated I2020T‐LRRK2.^12^ If the cellular milieu in the iPD brain (or peripheral immune cells) leads to a post‐translational modification of even wild‐type (WT) LRRK2, then we might expect to detect increased phosphorylation by isolated LRRK2 in our in vitro activity assay in the absence of a mutation.

In summary, we report here a significant difference between carriers and noncarriers in the intrinsic kinase activity of LRRK2 purified from PBMCs; however, our data highlight that there is an urgent need for more sensitive and accurate biomarkers of LRRK2 activity. In addition, analyses of samples from longitudinal collection of carefully phenotyped cohorts are required to follow up on our observation associating pSer935 LRRK2 with worse cognitive functioning.

## Author Roles

(1) Research Project: A. Conception, B. Organization, C. Execution; (2) Statistical Analysis: A. Design, B. Execution, C. Review and Critique; (3) Manuscript Preparation: A. Writing of the First Draft, B. Review and Critique.

K.M.: 1B, 1C, 3B

M.S.K.: 1B, 1C, 3B

C.L.: 1B

S.N.: 1C, 3B

N.L.: 1C

N.J.: 1C

K.K.: 1A, 1B, 3B

S.J.H.: 1A, 1B, 3B

M.A.S.B.: 1A, 1B, 3B

S.P.: 1A, 1B, 3B

U.J.K.: 1B, 3B

L.S.: 1B, 3B

R.N.A.: 1A, 1B, 1C, 2B, 2C, 3B

H.J.R.: 1A, 1B, 1C, 2A, 2B, 3A

## Financial Disclosures

U.J.K. has served on advisory boards for the American Parkinson Disease Association; has received honoraria from Iowa State University, The Parkinson's Foundation, and the Michael J. Fox Foundation for Parkinson's Research; and has received grants from NIH, The Parkinson's Foundation, the Michael J. Fox Foundation for Parkinson's Research, and the Jain Foundation. L.S. has served on advisory boards for AbbVie; has received honoraria from AbbVie and Sanofi; has received grants from the Michael J. Fox Foundation, EU, H2020, and SANTE Research Grants in Biomedical Sciences. R.N.A. has received consultation fees from Roche, Sanofi, Restorbio, and Janssen and has received grants from NIH, The Parkinson's Foundation, and the Michael J. Fox Foundation. H.J.R. has received grants from The Parkinson's Foundation, Michael J. Fox Foundation for Parkinson's Research, and The Parkinson's and Movement Disorder Foundation.

## Supporting information


**Appendix** S1: Supplementary Material

**Suppl. Figure 1** A) Schematic of the collection and storage protocols employed in this study. Whole blood was collected in Vacutainer ™ tubes containing either Heparin (green caps), Sodium Citrate (yellow caps). For PBMC isolation, all samples were diluted 1:1 in PBS and centrifuged in LeucoSep tubes containing Ficoll. Following washing, the cells collected in Heparin or Citrate tubes were re‐suspended in cryopreservation buffer containing 10% DMSO. Alternatively, cells from a second Heparin‐coated tube were simply washed and the cell pellet snap‐frozen in dry ice. In all PBMC isolation conditions, the cells were counted, and aliquoted into cryovial at a density of 3x10^6^ viable cells each. All samples were stored at ‐80°C until use. B) Schematic of LRRK2 indicating functional domains and location of epitopes for the antibodies used in this assay. Rabbit monoclonal (c41‐2; capture antibody) and mouse monoclonal (N241A; detection antibody) in tissue from mice deficient in LRRK2. A representative Western immunoblot detecting LRRK2 using both antibodies in wild type (WT) striatal or kidney extracts, striatum from LRRK2 knock out mice, or as a positive control, extracts from HEK293T cells over‐expressing human WT LRRK2. Both antibodies failed to detect a positive band for LRRK2 in striatal extracts from KO brain (lower panels).

**Suppl. Figure 2 Validation of novel LRRK2 sandwich ELISA.** A) Human recombinant full‐length WT LRRK2 was used to establish a calibration curve. Increasing amounts of rhLRRK2, in triplicate technical replicates, were processed by ELISA. Shown are representative plots from at least three biological replicates. The lower limit of detection is calculated as 2 standard deviations (SDs) greater than the mean of 20 blank wells processed identically as the calibration curve. B) The coefficient of variation was determined for “low” (0.6125 ng/ml) and “high” (5 ng/ml) spiked rhLRRK2 over several independent assays. C) The percent recovery of signal in sample matrix was estimated by spiking triplicate wells of rhLRRK2 at 0.6125 or 5 ng/ml in PBMC extract diluted 100X in TBST/BSA buffer, and performing the ELISA at least 3‐4 times. D) Variability in antibody performance was assessed by comparing multiple lots of the capture antibody (clone c41‐2) using increasing amounts of rhLRRK2. E) We compared the levels of LRRK2 expression, using our ELISA, in kidney or striatum from WT mice, as well as striatum from LRRK2‐KO mice. We detected robust LRRK2 signals in WT mouse striatum, and higher levels in WT kidney; however, we failed to detect a specific signal above background in the extracts of LRRK2‐KO striatum. F) Increasing amounts of striatal tissue from WT or KO mice were incubated in anti‐LRRK2 coated ELISA plates, and processed as before. Only in extracts of WT striatum did we detect a specific signal above background. G) We compared the expression in WT striatum and kidney with increasing amounts of protein extracts incubated in the plate. At all protein amounts, we detect higher levels of expression in kidney compared to striatum.

**Suppl. Figure 3 Validation of LRRK2 kinase activity assay**. WT LRRK2 over‐expressed in HEK293T cells was captured on ELISA plates pre‐coated with anti‐LRRK2 (c41‐2), and processed for *in vitro* kinase activity and total LRRK2 ELISA. Increasing amounts of protein extract containing over‐expressed LRRK2 lead to increased phosphorylation of LRRKtide (A) or NICtide (B) peptide substrates. WT (C) or G2019S (D) LRRK2 was purified on ELISA plates pre‐coated with anti‐LRRK2 (c41‐2), and processed for *in vitro* kinase activity in the presence of increasing concentrations of the kinase inhibitor GSK2578215A. ANOVA, Tukey post‐hoc comparisons; * p < 0.05, ** p < 0.01, *** p < 0.001.

**Suppl. Figure 4 Correlation between LRRK2 levels/activity and age.** A) LRRK2 levels (ng/ml) as measured by ELISA do not significantly differ between collection protocol or subject group. B) The mean age of subjects in the LRRK2+/PD+ and LRRK2+/PD‐ groups was compared; the age of affected carriers was significantly elevated compared to healthy carriers. *** p < 0.001. LRRK2 activity (C) and levels (D) and their correlation with subject age. We found no correlation in LRRK2 levels (ng/ml) in either LRRK2+ group with age (D); however, kinase activity was negatively correlated with age only in healthy carriers of the G2019S mutation (C).

**Suppl. Figure 5 Comparison of freshly isolated PBMCs vs cells isolated after delay**. From several healthy volunteers, 2 Heparin‐coated blood collection tubes were obtained. One tube was processed immediately for PBMC isolation, with the second tube kept at room temperature for 24h prior to PBMC isolation. We compared LRRK2 expression by Western immunoblotting (A) and ELISA (B), and find a marked reduction in LRRK2 levels in PBMCs isolated from blood samples left for 24h before isolation.

**Table 1** Demographics and LRRK2 activity among the participants by genotype and PD status
**Table 2**. Antibodies used in the ELISA‐based assays of LRRK2 function.
