# Supplementary material for: Elevated In Vitro Kinase Activity in Peripheral Blood Mononuclear Cells of Leucine‐Rich Repeat Kinase 2 G2019S Carriers: A Novel Enzyme‐Linked Immunosorbent Assay–Based Method
Source: Mov Disord. 2020 Jul 11;35(11):2095–100. doi: 10.1002/mds.28175 (PMC7754308; doi:10.1002/mds.28175)
Supplement: Supplementary file 1 — Appendix S1: Supplementary Material [file MDS-35-2095-s001.docx]

Elevated *in vitro* kinase activity in PBMCs of *LRRK2* G2019S carriers: a novel ELISA-based method

Katerina Melachroinou^a^; Min Suk. Kang^b^, Christopher Liong^b^, Sushma Narayan^b^, Najah Levers^b^, Neal Joshi^b^, Samantha J. Hutten^c^, Marco A. S. Baptista^c^, Shalini Padmanabhan^c^, Un Jung Kang^d^, Leonidas Stefanis^a,e^, Roy N. Alcalay^b^, Hardy J Rideout^a^*

^a^Division of Basic Neurosciences, Biomedical Research Foundation of the Academy of Athens, Athens, Greece; ^b^Department of Neurology, Columbia University, NY, USA; ^c^The Michael J. Fox Foundation for Parkinson’s Research, New York, NY; ^d^Department of Neurology, NYU Langone Health, New York, NY, ^e^Department of Neurology, University of Athens Medical School, Athens, Greece

**SUPPLEMENTARY MATERIAL**.

**Materials and Methods**.

*Study Participants*. Participants were divided to four groups based on their *LRRK2* G2019S and PD status. *LRRK2* G2019S testing was performed as previously described [1, 2]. Similarly, all participants were genotyped for 10 mutations and variants in *GBA* [1, 2]. At each study visit, participants were asked to give 4 tubes of whole blood (see schematic in Suppl. Fig. 1a), as well as urine (as described; [3, 4]). In this report, we describe the results of the various analyses of LRRK2 in the isolated PBMCs

*Sample Collection.* All samples were collected, processed and coded at the time of collection so that the analyses of the samples were performed by researchers blinded to the PD and mutation status. The study was approved by the institutional ethical review boards of both Columbia University (RNA) as well as Biomedical Research Foundation of the Academy of Athens (BRFAA; HJR), and all participants signed informed consent.

*PBMC Isolation*. PBMCs were isolated from whole blood using three different protocols; with two employing lithium heparin-coated Vacutainer collection tubes, and the last using sodium citrate Vacutainer collection tubes. Approximately 8-10 ml of whole blood, collected in the indicated vacuum tubes, were diluted 1:1 with phosphate-buffered saline (PBS). The diluted blood, from either of the blood collection vial types, was added to 50 ml Leucosep™ Tubes (Greiner Bio-One) which contain Ficoll-Paque PLUS (Millipore Sigma) and centrifuged at 1,000 g for 10 min at RT as per the manufacturer’s instructions. Three layers occur above the barrier: a plasma layer, the interphase consisting of PBMCs, and a small layer of Ficoll-Paque. Transfer the interphase white layer above the barrier, which contains the PBMCs, to a new conical tube and centrifuge at 250 g for 10 min in RT to get the cell pellets. Remove contaminating RBCs and platelets by washed twice in PBS, and gently resuspend with suitable amount of PBS or freezing medium consist of RPMI with 40% FBS and 10% DMSO for cell count. The PBMC cells were counted and deposited into aliquots (at 3x10^6^ viable cells/vial) for storage under the following conditions: Cells isolated in one of the heparin-coated tubes were resuspeneded in PBS and given a final centrifugation of 10,000 g for 10 min at 4°C, and the cell pellet was snap-frozen on dry ice before immediate storage at -80°C (sample “A”); cells isolated from the sodium citrate-containing tube (sample “B”), or from the second heparin-coated tube (sample “C”), were resuspended in a freezing medium consisting of RPMI with 40% FBS and 10% DMSO, and immediately stored at -80°C. Alternatively, parallel cells obtained from healthy volunteers were washed and resuspended in RPMI medium containing 40% FBS, if the cells were to be treated with LRRK2 kinase inhibitors. Cells were lysed in extraction buffer as indicated. For lysis, PBMCs were washed with ice-cold PBS, re-suspended in extraction buffer (50 mM Tris HCl pH 7.5; 1 mM EGTA; 0.27 M sucrose; and 1% Triton X-100; supplemented with protease & phosphatase inhibitor cocktail [Roche]), incubated on ice for 30 min and then centrifuged at 45,000 rcf for 1 h at 4°C. The supernatant was aliquoted and stored at -80 °C. It is possible that the Triton X-100 based lysis buffer would not completely extract all species of LRRK2 protein, and that some may remain in Triton-insoluble, SDS-soluble fractions, for example. In pilot studies during the validation phase of this study, we assessed other lysis buffers, including SDS-containing buffers, and found them incompatible with the ELISA conditions. Thus it remains possible that a small fraction of LRRK2, present in SDS-soluble fractions is not assessed by this method.

*Antibodies and reagents***.** See Suppl. Table 2 for the list of antibodies used in this study. Rabbit monoclonal anti-LRRK2 (total) and pS935-LRRK2 antibodies (clones c41-2, and UDD3; UDD2 for pS935) were purchased from Abcam (Cambridge, UK). Mouse monoclonal anti-LRRK2 (clone N241A/34) was from NeuroMab/Antibodies Incorporated (CA, USA), and conjugated to HRP using the Lightning-Link HRP conjugation kit (Expedeon; Heidelberg, DE). Anti-β-actin antibody used in Western immunoblotting analysis, for the evaluation of the equal loading, was purchased from Sigma (MO, USA). Rabbit anti-phospho-EZRIN/ERM and anti-pThr-X-Arg, used for the detection of LRRK2 phosphorylated peptide substrates, were from Cell Signaling (Cell Signaling Technology; MA, USA). HRP-conjugated anti-mouse and anti-rabbit secondary antibodies were purchased from DAKO. Biotin-LRRKtide and 6XHis-Nictide, model substrates of LRRK2 that were used in the ELISA-based kinase activity assays, were purchased from Innovagen (Lund, SE). As a control in the kinase reactions, GSK2578215A (Tocris, MN, USA), PF475 (Sigma; MO, USA), or MLi-2 (provided by Prof. Dario Alessi, University of Dundee), were used to block LRRK2 kinase activity.

*Plasmids.* Wild type (WT) LRRK2 cDNA with an N-terminal Flag epitope tag was used as described [5, 6]. Kinase inactive LRRK2 mutations, either D1994A or K1906R, or phosphorylation-deficient mutants, as indicated (e.g. S935A or S1292A), were introduced using site-directed mutagenesis (Quikchange Lightning; Agilent Technologies, CA, USA), and fully sequenced.

*Cell culture.* HEK293T cells were cultured in DMEM (Sigma; MO, USA) medium supplemented with 10% FBS, penicillin (100 U/ml), streptomycin (100 􏰂g/ml), and 2 mM L-glutamine. For the overexpression of Flag-tagged LRRK2, cells were transiently transfected in 6-well tissue culture plates using calcium phosphate:DNA precipitates. Cells were homogenized using lysis buffer as indicated and further analyzed for LRRK2 expression and kinase activity 72 h post transfection.

*Western Immunoblotting***.** For Western immunoblotting analysis, 100 μg of homogenized tissue and 15 μg of lysate from WT-LRRK2 overexpressing cells were separated by SDS-PAGE. For assessment of PBMCs, 10 μg of total extract was used. Proteins were separated by SDS-PAGE and transferred to nitrocellulose membranes, followed by blocking in 5% BSA/TBST. Primary antibodies against LRRK2 (rabbit monoclonal clone c41-2, 1:5000; mouse monoclonal clone N241A/34, 1:1000; rabbit monoclonal clone UDD3, 1:4000; phospho-S935, clone UDD2, 1:4000); and β-actin (1:5000) were diluted in 5% BSA/TBST and incubated overnight at 4°C. The next day, the membranes were washed and HRP-conjugated anti rabbit or mouse antibodies diluted in 5% non fat milk were applied to the membranes for 1h at room temperature. Bands were visualized using Dura-West ECL substrate (Pierce/Thermo Scientific).

*Animals*. Tissue homogenates from two month-old male C57BL/6 LRRK2 knock-out mice (KO) lacking exon 41 that encodes the activation hinge of the kinase domain of LRRK2 [7], as well as wild-type (WT) littermates were used to evaluate the specificity of the antibodies used in the LRRK2-specific ELISA. All animal procedures using LRRK2 KO C57BL/6 mice were approved by the Mayo Clinic Institutional Animal Care and Use Committee (Jacksonville, USA) and were in accordance with the National Institute of Health Guide for the Care and Use of Laboratory Animals. WT animals were housed in the animal facility of the BRFAA in a room with a controlled light-dark cycle (12 hours light-12 hours dark) and free access to food and water. Animal procedures were performed under the approval of the Ethical Committee for Use of Laboratory Animals at BRFAA. Mice were deeply anesthetized by an overdose of pentobarbital and perfused transcardially with PBS, to avoid blood contamination; brains were harvested and dissected on ice to obtain the striata and ventral midbrain regions. Kidneys from WT mice were also isolated. All animals were processed in a similar manner. Tissue was dissected and homogenized in Tween-20 extraction buffer (150 mM NaCl; 20 mM Tris, pH 7.6; 0.5% Tween-20; 1 mM EDTA, supplemented with protease & phosphatase inhibitor cocktails [Roche]). Following 3 cycles of sonication (7 sec, 33% amplitude, 1 min interval on ice), the homogenate was incubated on ice for 30 min and centrifuged at 45,000 rcf for 1 h at 4°C. The supernatant was stored at -80 °C.

*Endogenous LRRK2 ELISA and in vitro kinase activity.* Each ELISA plate (Corning Costar) was coated overnight at RT with rabbit monoclonal anti-LRRK2 (clone c41-2) in 100 mM NaHCO_3_, pH 9.3-9.6. Before protein binding, the plates were washed 4 times with ELISA wash buffer (50 mM Tris, pH 7.4; 150 mM NaCl; 0.1% Tween-20). To allow antigen binding, plates were incubated at 37 °C for 2 h 30 min. After washing 4 times with ELISA wash buffer, the wells were incubated with HRP-conjugated mouse monoclonal anti-LRRK2 antibody (diluted in TBS-T/1% BSA) for 1 h at RT. The wells were washed and chemiluminescent substrate (Thermo Scientific) was added to each well, for 5 min at RT. Alternatively, levels of phosphorylated LRRK2 were normalized to parallel wells processed for the total LRRK2 levels as described above. Following capture of LRRK2, the wells were washed 3 times with ELISA wash buffer, followed by 2 washes with kinase reaction buffer (20 mM Tris, pH 7.5; 20 mM NaCl; 10 mM MgCl_2_; 2 mM DTT). Kinase reaction buffer containing 100 µM ATP, 5 µM of LRRK2 substrate [biotinylated LRRKtide (biotin-LRRKtide) or 6His-NICtide (Innovagen; Lund, SE)], and protease/phosphatase inhibitors (Roche) was added to each well and incubated for 30 min at 30°C. At the end of the reaction, the mixture was removed and diluted in ice-cold EDTA-containing binding buffer (50 mM Tris, pH 7.6; 150 mM NaCl; 0.5% NP-40; 20 mM EDTA) and 50 μl of the diluted reaction was added to streptavidin-coated 96-well plates. When 6His-NICtide was used as a substrate, the reaction was diluted with EDTA-free binding buffer (50 mM Tris, pH 7.6; 150 mM NaCl; 0.5% NP-40) and added to Nickel-coated 96-well plates (Thermo Scientific; PA, USA). The plates were incubated at 37°C for 1 h, followed by 4 washes with ELISA wash buffer. Then, anti-phospho-ERM (diluted in TBS-T/1% BSA, Cell Signaling) or anti-phospho-Thr-X-Arg (diluted in TBS-T/1% BSA, Cell Signaling) were added to the wells for 1 h at RT, for the detection of phosphorylated biotin-LRRKtide or phosphorylated 6His-NICtide, respectively. Following 4 washes, the wells were incubated with HRP-conjugated anti-rabbit secondary antibody (diluted in TBS-T/1% BSA, DAKO) for 1 h at RT. Finally, the wells were washed and incubated for 5 min at RT with chemiluminescent substrate (Pierce/Thermo Scientific; MA, USA). As a control, wells in which no protein was bound were treated identically at each subsequent step, to confirm the selectivity of the phospho-antibodies. Additionally, to evaluate the specificity of the kinase activity assay 1 µM of the LRRK2 kinase inhibitor (MLi-2) was added during the kinase reaction. In-well kinase activity is presented as the ratio of the anti-phospho-EZRIN/ERM (phospho-LRRKtide) or anti-phospho-Thr-X-Arg (phospho-NICtide) chemiluminescence value to signal of total LRRK2 bound to the respective well.

**Results & Discussion**.

*Antibody Specificity.* The two rabbit monoclonal antibodies, c41-2 and UDD3, recognize epitopes within the namesake LRR domain and N-terminal domain, respectively (see Schematic in Suppl. Fig. 1b, and Supplementary Table 2). Conversely, the mouse monoclonal antibody (clone N241A) maps to the C-terminal region (Suppl. Fig. 1b). In order to test the specificity of the above anti-LRRK2 antibodies, striatal tissue from WT C57/BL6 mice and LRRK2-KO littermates was analyzed for the presence of LRRK2. As shown in Suppl. Fig. 1b, the clones c41-2 and N241A show immunoreactivity with LRRK2 in WT striatal homogenate, which was absent in KO protein extract. Additionally, since it is well established that kidneys are enriched in LRRK2, kidney homogenates from WT animals were used as an internal control for the detection of endogenous LRRK2. Finally, as a further positive control we used cell lysate from HEK 293T overexpressing Flag-tagged WT LRRK2. The rabbit monoclonal, clone c41-2, and N241A/34 mouse monoclonal antibodies were chosen for the development of our ELISA for measuring the levels of the endogenous LRRK2, as capture and detection antibodies, respectively.

To establish the lower limit of detection (LLOD), or sensitivity of the assay, we measured 12 replicate zero standards, and the calculated OD two standard deviations above this mean was input into the standard curve regression equation to obtain the theoretical LLOD of ~ 0.05 ng/ml (Suppl. Fig. 2a). To determine the precision of this assay, we performed a series of additional measurements to assess: a) precision, b) antibody lot variability, c) assay drift, and d) spike and recovery (to establish the presence of potential matrix effects). We performed several identical standard curve assays with rhLRRK2 over several days using separate batches of coated ELISA plates. The coefficient of variation between two points of the standard curve (“low” spiked standard of 0.6125 ng/ml; and “high” spiked standard of 5 ng/ml) across all the individual measurements ranged between 3-8% (Suppl. Fig. 2b). To assess whether significant differences in antibody lot would impact the precision of the ELISA, we prepared three independent ELISA plates coated (at identical concentrations) with different lots of anti-LRRK2 (clone c41-2) antibodies obtained from Abcam. Multiple points on the standard curve were loaded in each coated well and processed identically using the same N241A/34-HRP LRRK2 antibody as a detection antibody. As is evident from the resulting curves, the different lots of antibody did not alter the linearity or sensitivity of the assay (Suppl. Fig. 2c). To assess assay drift, whether the position in the ELISA plate influenced the chemiluminescence OD obtained, we assessed multiple points on the standard curve under identical conditions at different places within the ELISA plate. The CV obtained across the different positions was less than 10% (not shown).

A critical aspect of the assay is to ensure that there is a minimal sample matrix effect. It is necessary to rule out the possibility that components of the extraction buffer or cell extract itself do not interfere with detection of the analyte, in this case LRRK2. In contrast to ELISA’s assessing levels of α-synuclein or cytokine levels in clinical biofluids such as CSF or serum, the LRRK2 ELISA presented here is optimized for the detection of LRRK2 in cellular/tissue extracts, such as brain, fibroblast or PBMC extracts, although we predict that there will be a similar performance for other sources of LRRK2 (e.g. LRRK2 present in exosomes, or extra-cellular vesicles [EVs]). As such, we set to determine the existence of potential matrix effects specific for these tissue sources. This can be assessed in two ways, by spiking known amounts of rhLRRK2 into the cell extract being measured and determining the % recovery. If a significant matrix effect exists, the % recovery of spiked rhLRRK2 is suppressed. Secondly, if % recovery is low, different dilutions of the sample in standard diluent (TBST/1% BSA) can be applied to minimize the matrix effect. In diluted PBMC cell extract, we spiked 2 different amounts of rhLRRK2 and performed the ELISA as described using anti-LRRK2 (N241A/34-HRP) as the detection antibody. Our pilot studies indicated that as the amount of cell extract and spiked rhLRRK2 increased, the % recovery decreased (not shown), suggesting that the amount of LRRK2 present in the spiked sample fell outside the dynamic range of the assay; or, that a component of the cell extract or lysis buffer was interfering with the detection of LRRK2. Preparation of cell extracts in an alternative lysis buffer (containing 0.5% NP-40 and glycerol instead of 1% Triton X-100 and sucrose) only moderately suppressed the signal suggesting that the matrix effect was present in the cell extract (not shown).

We assessed the specificity of the novel LRRK2 ELISA in whole brain tissue from WT or LRRK2 deficient mice. As depicted in Suppl. Fig. 2 (e, f), LRRK2 levels are not detectable in striatal homogenates from KO mice, while in the respective homogenates from WT littermates LRRK2 concentration is estimated to be 0.40 ± 0.12 ng/ml. In accord with previous reports showing markedly higher LRRK2 expression levels, our LRRK2 ELISA measured significantly higher LRRK2 levels in the WT kidney homogenates (1.32 ± 0.26 ng/ml), compared to WT striatum. In addition, we loaded increasing amounts (5, 10, 50 and 150 μg) of tissue homogenates (diluted 100X in standard diluent as determined above) from WT striatum and kidneys and measured LRRK2 levels. In WT kidney extracts, LRRK2 levels were proportionally increased in all cases. However, in WT striatal homogenates we were able to detect an increase in expression levels starting from 50μg, since in 5 and 10 μg of lysates, LRRK2 levels were below detection limit (Suppl. Fig. 2g). LRRK2 was not detected in KO striatal tissue under all conditions. Collectively, our results show that our novel LRRK2 ELISA can specifically detect and quantify endogenous LRRK2 from a variety of tissue and cell types, including rodent and human.

*An ELISA-based assay for assessment of LRRK2 kinase* activity. Following binding to the LRRK2-coated ELISA wells, a kinase reaction mix containing either biotin-LRRKtide or 6His-Nictide, two different artificial LRRK2 substrates, is added to the wells. Following the reaction, the supernatant of the reaction is removed and added to either streptavidin-coated or Nickel-coated multi-well plates, respectively. We measured LRRK2 kinase activity by measuring the phosphorylation of the substrates with pLRRKtide or pThr-X-Arg antibodies, respectively. As shown in Suppl. Fig. 3, (a, b), the chemiluminescent signal of the phosphorylated substrates increases as the amount of protein loaded is increased. As a control for the specificity of the kinase reaction, WT or G2019S-LRRK2 was purified from HEK293T cells on anti-LRRK2 (c41-2) coated ELISA plates and the kinase reaction was performed in the presence of increasing amounts of the LRRK2 inhibitor GSK2578215A. We find a dose-dependent loss of LRRKtide phosphorylation by WT and G2019S-LRRK2 when the reaction is performed in the presence of the inhibitor; however, G2019S-LRRK2 exhibited a slight loss of sensitivity to inhibition compared to WT LRRK2 (Suppl. Fig. 3 c, d).

In comparing the different isolation and storage methods, we found no significant difference between the various methods in overall detection of LRRK2 activity by ELISA (not shown). Kinase activity in *LRRK2* G2019S mutation carriers PBMCs isolated using the other approaches (“A” and “C” samples), showed a trend toward higher *in vitro* activity, but without statistical significance (not shown). It is unclear why there was an apparent effect of the collection tube on the downstream *in vitro* kinase assay, with citrate; however, this observation is in agreement with previous findings indicating that PBMCs isolated in CPT collection tubes exhibit greater detectable activity in comparison to EDTA-containing tubes (M. Liu; personal communication). Improvements in the sensitivity of the assays can also help clarify these questions (e.g. exploring other detection antibodies); as in several of the samples, the chemiluminescence signal did not reach the threshold (2X the signal of the blank) for total LRRK2 levels. In these specific samples, we could detect robust *in vitro* kinase activity and pS935-LRRK2 levels, however the low specific signal in the total LRRK2 ELISA prevented the normalization of the activity or pS935 assay, resulting in some samples being omitted from the final analyses.

*Phosphorylation of LRRK2 at Ser935*. To determine the linear range of the pS935-LRRK2 ELISA, we incubated increasing amounts of the calibrator recombinant full-length LRRK2 protein in wells pre-coated with anti-LRRK2 (clone c41-2), and performed parallel ELISA’s using either total LRRK2 (N241A) or pS935-LRRK2 antibodies as detector reagents. In both cases, a linear response was obtained starting approximately at 0.156 ng/ml LRRK2 through 10 ng/ml (Fig. 2a); with an R^2^ value for total and pS935-LRRK2 of 0.99147 and 0.99845, respectively. To confirm whether our ELISA can detect changes in pS935-LRRK2 levels that have been reported in the recent literature, we measured pS935-LRRK2 by ELISA in extracts of HEK293T cells over-expressing WT or disease-linked mutant LRRK2, and total LRRK2 in parallel plates. As has been reported, both R1441C- and I2020T-LRRK2 lead to a significant decrease in pS935 levels, as a proportion of total LRRK2 (Fig. 2b), whereas G2019S did not have an effect on pS935-LRRK2 levels in any direction. As confirmation, S935A-LRRK2 expressing extracts or MLi-2 treated cells, show a robust loss of pS935-LRRK2 levels in comparison to WT-LRRK2 expressing cells (Fig. 2b).

*Clinical correlates with LRRK2 levels and phosphorylation & considerations for biomarker studies.* An important element in establishing the optimal conditions for a biomarker assay is to also assess and validate the quality and stability of the samples to be measured. In a large multi-centre biomarker study, it would be expected that in some cases the blood samples would be sent to a central site for processing and storage. To approximate this delay, and assess the performance of the assay in comparison to freshly isolated samples, we collected whole blood from 10 healthy volunteers using Heparin-coated Vacutainer tubes. Two samples were collected in parallel; with one sample processed immediately for the isolation of PBMCs, and the second sample left for 24hr at room temperature before isolation of the PBMCs. We compared total LRRK2 levels by Western immunoblot and ELISA for each sample pair (fresh vs. 24hr delay). In Suppl. Fig. 5 (a, b), a representative immunoblot and plot of LRRK2 levels from ELISA are shown. Surprisingly, we find a dramatic loss of expression of LRRK2 in samples isolated following a 24hr delay at room temperature. This indicates that processing of the samples (i.e. isolation of PBMCs) should be performed as soon as possible after the blood draw in order to preserve LRRK2 expression levels.

**Supplemental Figure Legends.**

**Suppl. Figure 1**. A) Schematic of the collection and storage protocols employed in this study. Whole blood was collected in Vacutainer ^TM^ tubes containing either Heparin (green caps), Sodium Citrate (yellow caps). For PBMC isolation, all samples were diluted 1:1 in PBS and centrifuged in LeucoSep tubes containing Ficoll. Following washing, the cells collected in Heparin or Citrate tubes were re-suspended in cryopreservation buffer containing 10% DMSO. Alternatively, cells from a second Heparin-coated tube were simply washed and the cell pellet snap-frozen in dry ice. In all PBMC isolation conditions, the cells were counted, and aliquoted into cryovial at a density of 3x10^6^ viable cells each. All samples were stored at -80˚C until use. B) Schematic of LRRK2 indicating functional domains and location of epitopes for the antibodies used in this assay. Rabbit monoclonal (c41-2; capture antibody) and mouse monoclonal (N241A; detection antibody) in tissue from mice deficient in LRRK2. A representative Western immunoblot detecting LRRK2 using both antibodies in wild type (WT) striatal or kidney extracts, striatum from LRRK2 knock out mice, or as a positive control, extracts from HEK293T cells over-expressing human WT LRRK2. Both antibodies failed to detect a positive band for LRRK2 in striatal extracts from KO brain (lower panels).

**Suppl. Figure 2.** **Validation of novel LRRK2 sandwich ELISA.** A) Human recombinant full-length WT LRRK2 was used to establish a calibration curve. Increasing amounts of rhLRRK2, in triplicate technical replicates, were processed by ELISA. Shown are representative plots from at least three biological replicates. The lower limit of detection is calculated as 2 standard deviations (SDs) greater than the mean of 20 blank wells processed identically as the calibration curve. B) The coefficient of variation was determined for “low” (0.6125 ng/ml) and “high” (5 ng/ml) spiked rhLRRK2 over several independent assays. C) The percent recovery of signal in sample matrix was estimated by spiking triplicate wells of rhLRRK2 at 0.6125 or 5 ng/ml in PBMC extract diluted 100X in TBST/BSA buffer, and performing the ELISA at least 3-4 times. D) Variability in antibody performance was assessed by comparing multiple lots of the capture antibody (clone c41-2) using increasing amounts of rhLRRK2. E) We compared the levels of LRRK2 expression, using our ELISA, in kidney or striatum from WT mice, as well as striatum from LRRK2-KO mice. We detected robust LRRK2 signals in WT mouse striatum, and higher levels in WT kidney; however, we failed to detect a specific signal above background in the extracts of LRRK2-KO striatum. F) Increasing amounts of striatal tissue from WT or KO mice were incubated in anti-LRRK2 coated ELISA plates, and processed as before. Only in extracts of WT striatum did we detect a specific signal above background. G) We compared the expression in WT striatum and kidney with increasing amounts of protein extracts incubated in the plate. At all protein amounts, we detect higher levels of expression in kidney compared to striatum.

**Suppl. Figure 3.** **Validation of LRRK2 kinase activity assay**. WT LRRK2 over-expressed in HEK293T cells was captured on ELISA plates pre-coated with anti-LRRK2 (c41-2), and processed for *in vitro* kinase activity and total LRRK2 ELISA. Increasing amounts of protein extract containing over-expressed LRRK2 lead to increased phosphorylation of LRRKtide (A) or NICtide (B) peptide substrates. WT (C) or G2019S (D) LRRK2 was purified on ELISA plates pre-coated with anti-LRRK2 (c41-2), and processed for *in vitro* kinase activity in the presence of increasing concentrations of the kinase inhibitor GSK2578215A. ANOVA, Tukey post-hoc comparisons; * p<0.05, ** p<0.01, *** p<0.001.

**Suppl. Figure 4. Correlation between LRRK2 levels/activity and age.** A) LRRK2 levels (ng/ml) as measured by ELISA do not significantly differ between collection protocol or subject group. B) The mean age of subjects in the LRRK2+/PD+ and LRRK2+/PD- groups was compared; the age of affected carriers was significantly elevated compared to healthy carriers. *** p<0.001. LRRK2 activity (C) and levels (D) and their correlation with subject age. We found no correlation in LRRK2 levels (ng/ml) in either LRRK2+ group with age (D); however, kinase activity was negatively correlated with age only in healthy carriers of the G2019S mutation (C).

**Suppl. Figure 5.** **Comparison of freshly isolated PBMCs vs cells isolated after delay**. From several healthy volunteers, 2 Heparin-coated blood collection tubes were obtained. One tube was processed immediately for PBMC isolation, with the second tube kept at room temperature for 24h prior to PBMC isolation. We compared LRRK2 expression by Western immunoblotting (A) and ELISA (B), and find a marked reduction in LRRK2 levels in PBMCs isolated from blood samples left for 24h before isolation.

**References.**

1. Alcalay, R.N., F. Hsieh, E. Tengstrand, et al., *Higher Urine bis(Monoacylglycerol)Phosphate Levels in LRRK2 G2019S Mutation Carriers: Implications for Therapeutic Development.* Mov Disord, 2019.

2. Alcalay, R.N., O.A. Levy, C.C. Waters, et al., *Glucocerebrosidase activity in Parkinson's disease with and without GBA mutations.* Brain, 2015. **138**(Pt 9): p. 2648-58.

3. Fraser, K.B., M.S. Moehle, R.N. Alcalay, A.B. West, and L.C. Consortium, *Urinary LRRK2 phosphorylation predicts parkinsonian phenotypes in G2019S LRRK2 carriers.* Neurology, 2016. **86**(11): p. 994-9.

4. Fraser, K.B., A.B. Rawlins, R.G. Clark, et al., *Ser(P)-1292 LRRK2 in urinary exosomes is elevated in idiopathic Parkinson's disease.* Mov Disord, 2016.

5. Antoniou, N., D. Vlachakis, A. Memou, et al., *A motif within the armadillo repeat of Parkinson's-linked LRRK2 interacts with FADD to hijack the extrinsic death pathway.* Sci Rep, 2018. **8**(1): p. 3455.

6. Ho, C.C., H.J. Rideout, E. Ribe, C.M. Troy, and W.T. Dauer, *The Parkinson disease protein leucine-rich repeat kinase 2 transduces death signals via Fas-associated protein with death domain and caspase-8 in a cellular model of neurodegeneration.* J Neurosci, 2009. **29**(4): p. 1011-6.

7. Hinkle, K.M., M. Yue, B. Behrouz, et al., *LRRK2 knockout mice have an intact dopaminergic system but display alterations in exploratory and motor co-ordination behaviors.* Mol Neurodegener, 2012. **7**: p. 25.
