# Supplementary figures and images for: Elevated In Vitro Kinase Activity in Peripheral Blood Mononuclear Cells of Leucine‐Rich Repeat Kinase 2 G2019S Carriers: A Novel Enzyme‐Linked Immunosorbent Assay–Based Method
Source: Mov Disord. 2020 Jul 11;35(11):2095–100. doi: 10.1002/mds.28175 (PMC7754308; doi:10.1002/mds.28175)

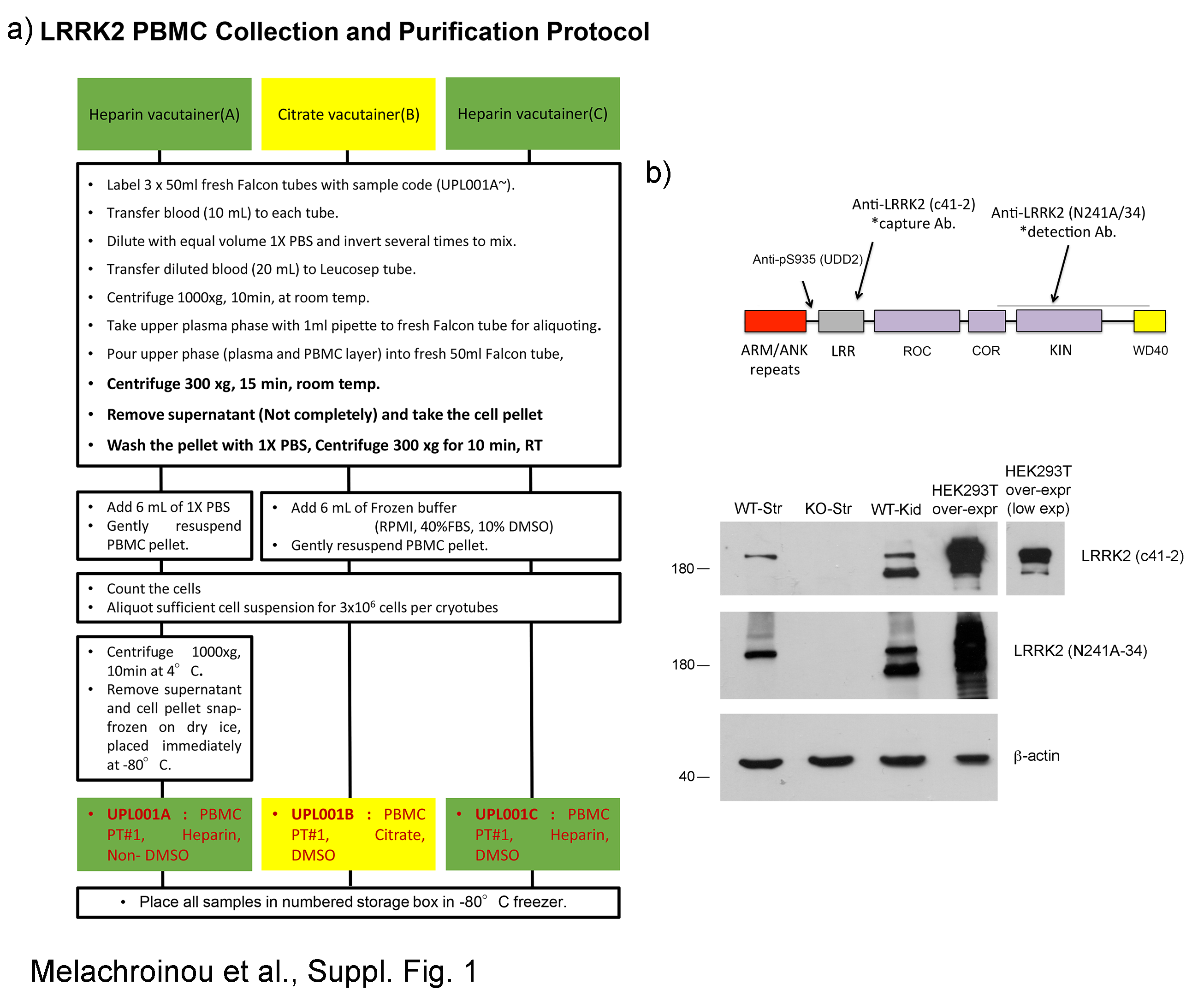

Supplement: Supplementary file 2 — Suppl. Figure 1 A) Schematic of the collection and storage protocols employed in this study. Whole blood was collected in Vacutainer ™ tubes containing either Heparin (green caps), Sodium Citrate (yellow caps). For PBMC isolation, all samples were diluted 1:1 in PBS and centrifuged in LeucoSep tubes containing Ficoll. Following washing, the cells collected in Heparin or Citrate tubes were re‐suspended in cryopreservation buffer containing 10% DMSO. Alternatively, cells from a second Heparin‐coated tube were simply washed and the cell pellet snap‐frozen in dry ice. In all PBMC isolation conditions, the cells were counted, and aliquoted into cryovial at a density of 3x10^6^ viable cells each. All samples were stored at ‐80°C until use. B) Schematic of LRRK2 indicating functional domains and location of epitopes for the antibodies used in this assay. Rabbit monoclonal (c41‐2; capture antibody) and mouse monoclonal (N241A; detection antibody) in tissue from mice deficient in LRRK2. A representative Western immunoblot detecting LRRK2 using both antibodies in wild type (WT) striatal or kidney extracts, striatum from LRRK2 knock out mice, or as a positive control, extracts from HEK293T cells over‐expressing human WT LRRK2. Both antibodies failed to detect a positive band for LRRK2 in striatal extracts from KO brain (lower panels). [file MDS-35-2095-s002.tif]

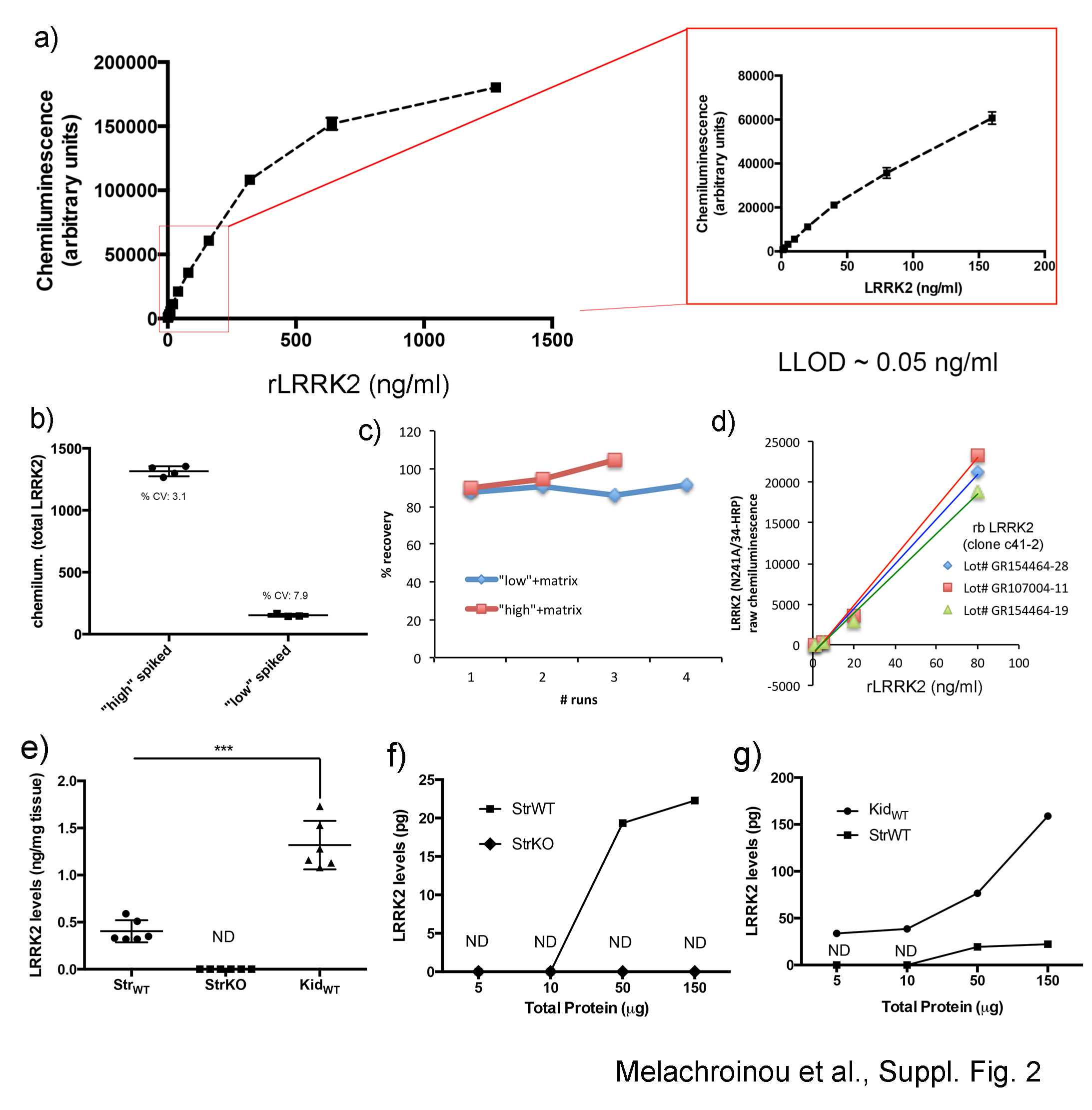

Supplement: Supplementary file 3 — Suppl. Figure 2 Validation of novel LRRK2 sandwich ELISA. A) Human recombinant full‐length WT LRRK2 was used to establish a calibration curve. Increasing amounts of rhLRRK2, in triplicate technical replicates, were processed by ELISA. Shown are representative plots from at least three biological replicates. The lower limit of detection is calculated as 2 standard deviations (SDs) greater than the mean of 20 blank wells processed identically as the calibration curve. B) The coefficient of variation was determined for “low” (0.6125 ng/ml) and “high” (5 ng/ml) spiked rhLRRK2 over several independent assays. C) The percent recovery of signal in sample matrix was estimated by spiking triplicate wells of rhLRRK2 at 0.6125 or 5 ng/ml in PBMC extract diluted 100X in TBST/BSA buffer, and performing the ELISA at least 3‐4 times. D) Variability in antibody performance was assessed by comparing multiple lots of the capture antibody (clone c41‐2) using increasing amounts of rhLRRK2. E) We compared the levels of LRRK2 expression, using our ELISA, in kidney or striatum from WT mice, as well as striatum from LRRK2‐KO mice. We detected robust LRRK2 signals in WT mouse striatum, and higher levels in WT kidney; however, we failed to detect a specific signal above background in the extracts of LRRK2‐KO striatum. F) Increasing amounts of striatal tissue from WT or KO mice were incubated in anti‐LRRK2 coated ELISA plates, and processed as before. Only in extracts of WT striatum did we detect a specific signal above background. G) We compared the expression in WT striatum and kidney with increasing amounts of protein extracts incubated in the plate. At all protein amounts, we detect higher levels of expression in kidney compared to striatum. [file MDS-35-2095-s003.tif]

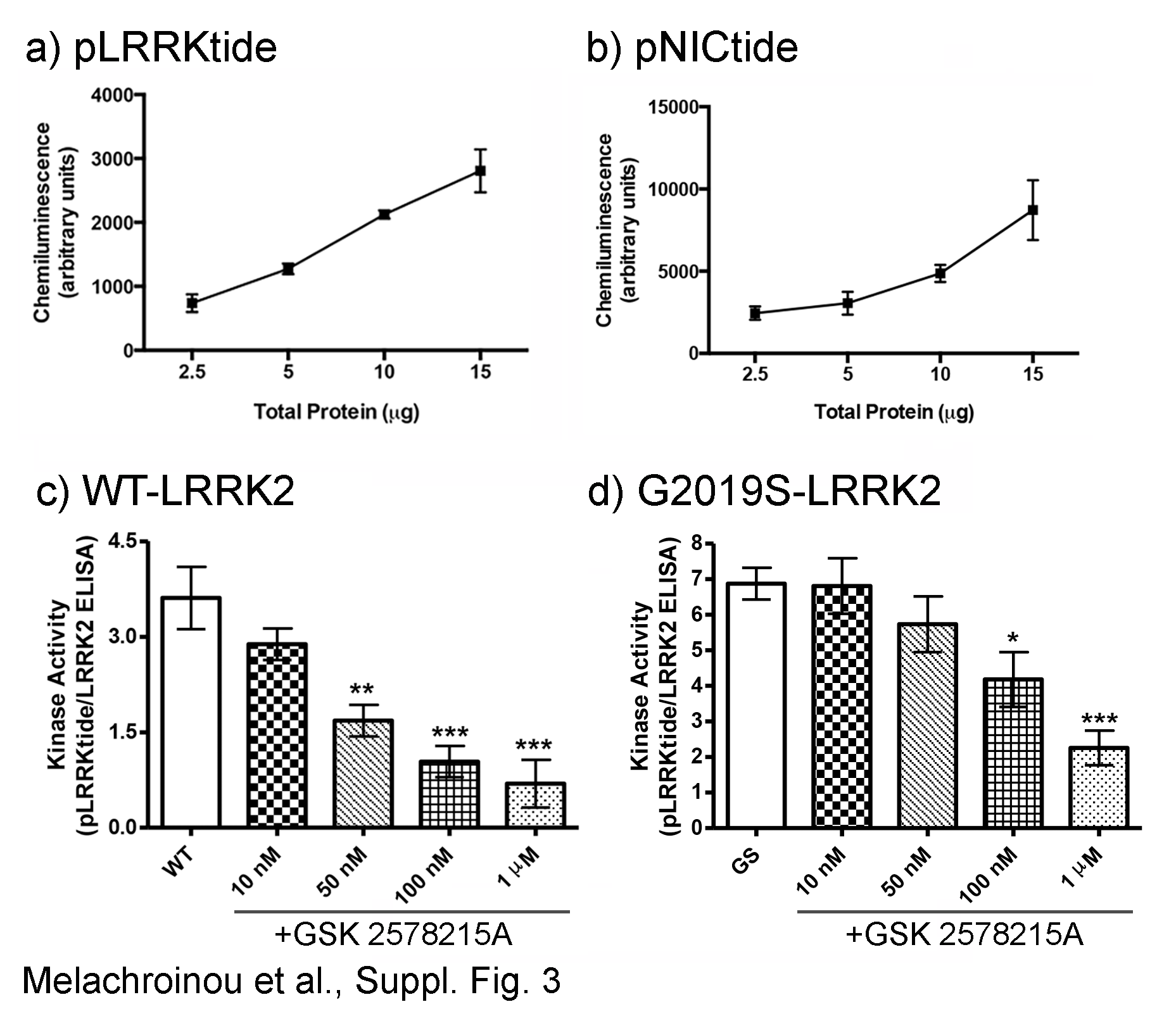

Supplement: Supplementary file 4 — Suppl. Figure 3 Validation of LRRK2 kinase activity assay. WT LRRK2 over‐expressed in HEK293T cells was captured on ELISA plates pre‐coated with anti‐LRRK2 (c41‐2), and processed for in vitro kinase activity and total LRRK2 ELISA. Increasing amounts of protein extract containing over‐expressed LRRK2 lead to increased phosphorylation of LRRKtide (A) or NICtide (B) peptide substrates. WT (C) or G2019S (D) LRRK2 was purified on ELISA plates pre‐coated with anti‐LRRK2 (c41‐2), and processed for in vitro kinase activity in the presence of increasing concentrations of the kinase inhibitor GSK2578215A. ANOVA, Tukey post‐hoc comparisons; * p < 0.05, ** p < 0.01, *** p < 0.001. [file MDS-35-2095-s004.tif]

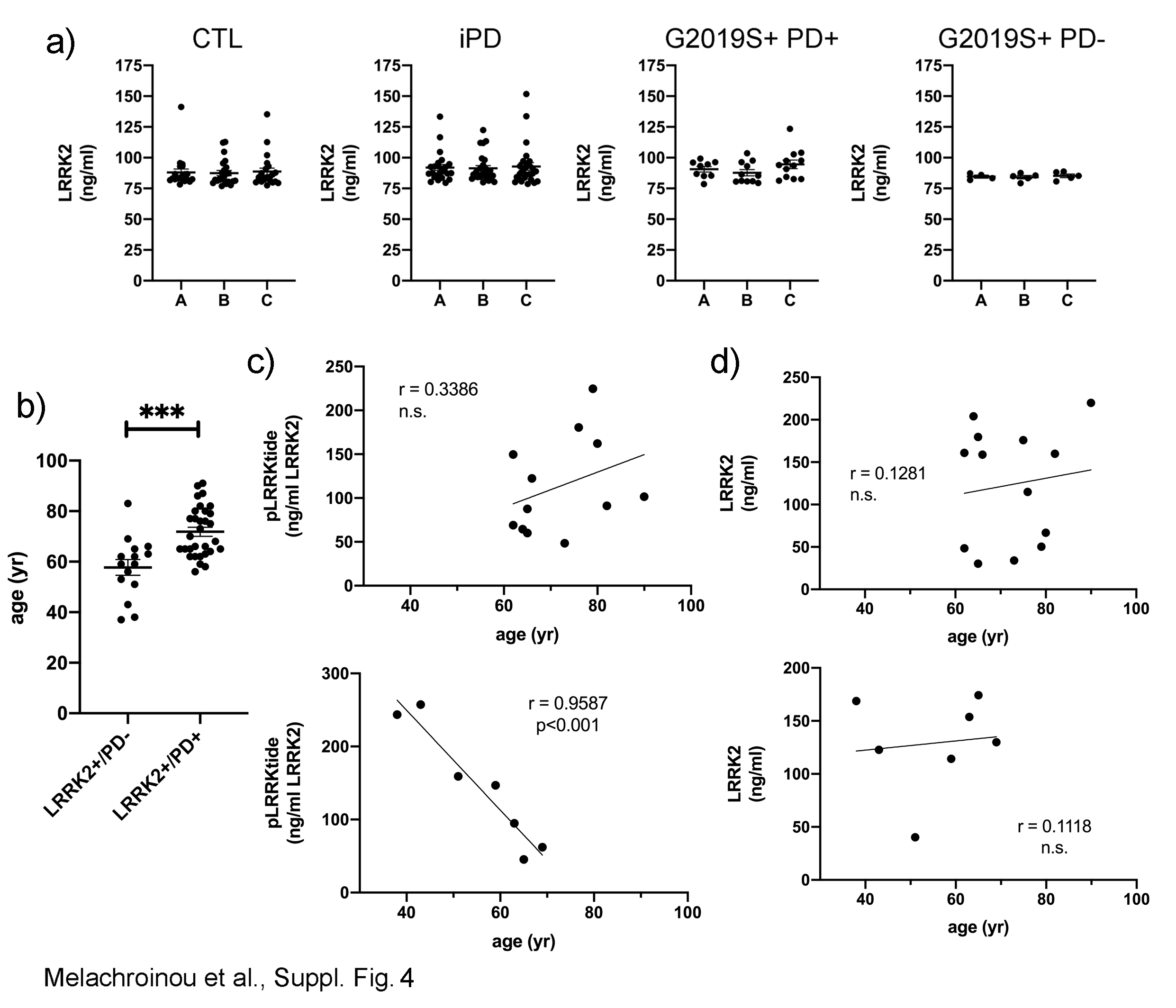

Supplement: Supplementary file 5 — Suppl. Figure 4 Correlation between LRRK2 levels/activity and age. A) LRRK2 levels (ng/ml) as measured by ELISA do not significantly differ between collection protocol or subject group. B) The mean age of subjects in the LRRK2+/PD+ and LRRK2+/PD‐ groups was compared; the age of affected carriers was significantly elevated compared to healthy carriers. *** p < 0.001. LRRK2 activity (C) and levels (D) and their correlation with subject age. We found no correlation in LRRK2 levels (ng/ml) in either LRRK2+ group with age (D); however, kinase activity was negatively correlated with age only in healthy carriers of the G2019S mutation (C). [file MDS-35-2095-s005.tif]

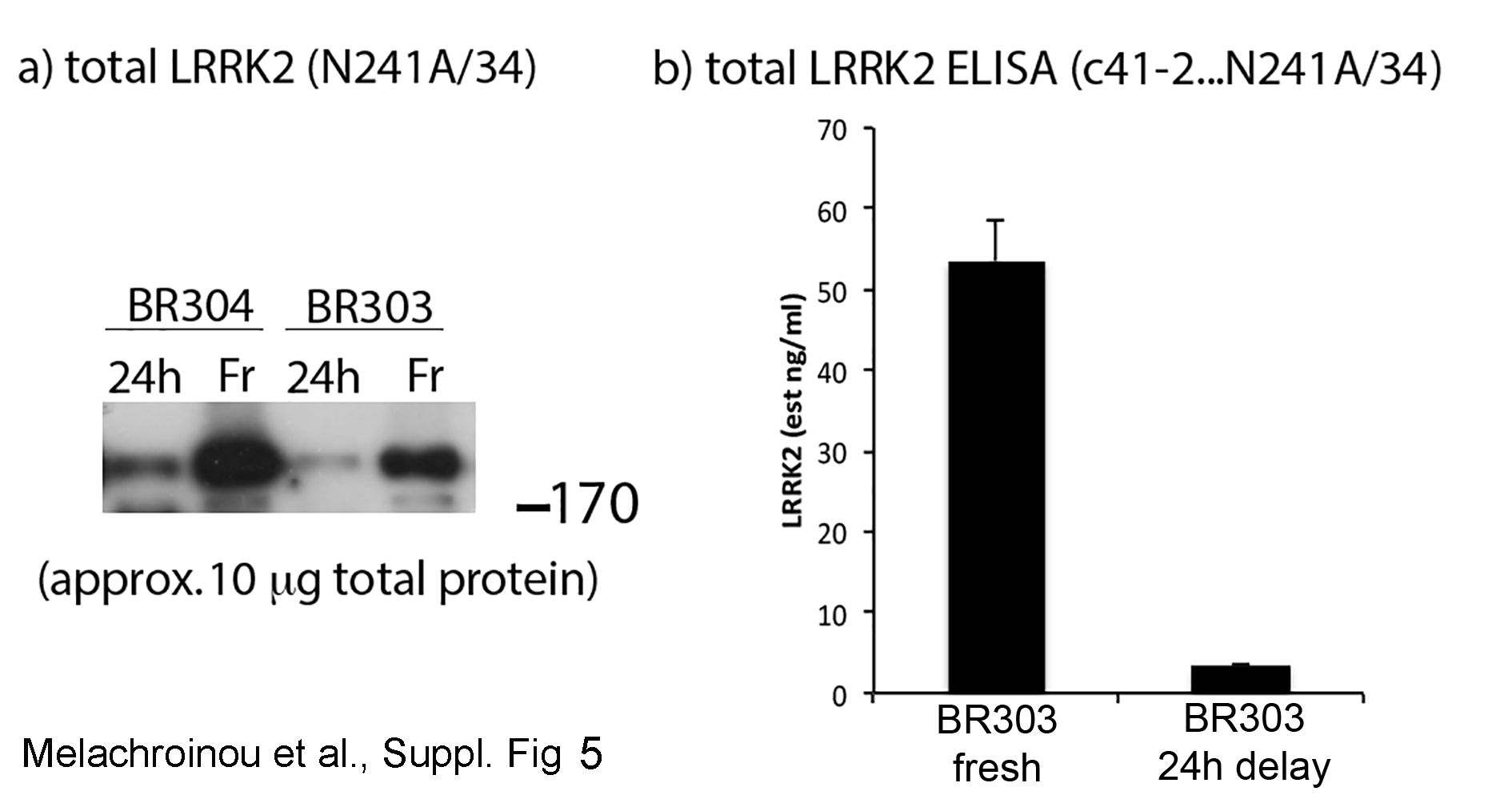

Supplement: Supplementary file 6 — Suppl. Figure 5 Comparison of freshly isolated PBMCs vs cells isolated after delay. From several healthy volunteers, 2 Heparin‐coated blood collection tubes were obtained. One tube was processed immediately for PBMC isolation, with the second tube kept at room temperature for 24h prior to PBMC isolation. We compared LRRK2 expression by Western immunoblotting (A) and ELISA (B), and find a marked reduction in LRRK2 levels in PBMCs isolated from blood samples left for 24h before isolation. [file MDS-35-2095-s006.tif]
