# Supplementary material for: Elevated In Vitro Kinase Activity in Peripheral Blood Mononuclear Cells of Leucine‐Rich Repeat Kinase 2 G2019S Carriers: A Novel Enzyme‐Linked Immunosorbent Assay–Based Method
Source: Mov Disord. 2020 Jul 11;35(11):2095–100. doi: 10.1002/mds.28175 (PMC7754308; doi:10.1002/mds.28175)
Supplement: Supplementary file 7 — Table 1 Demographics and LRRK2 activity among the participants by genotype and PD status Table 2. Antibodies used in the ELISA‐based assays of LRRK2 function. [file MDS-35-2095-s007.docx]

Table 1: Demographics and LRRK2 activity among the participants by genotype and PD status

|  | **PD+*LRRK2+***  (n=14) | **PD+*LRRK2-***  (n=35) | **PD-*LRRK2+***  (n=11) | ***PD-LRRK2-***  (n=27) | **p-value** |
| --- | --- | --- | --- | --- | --- |
| **Age mean, (SD)** | 73.1 (8.5) | 67.6 (9.1) | 58.9 (12.4) | 68.1 (8.5) | 0.004 |
| **Age-at onset mean, (SD)** | 61.7 (10.6) | 60.8 (11.2) | n/a | n/a | 0.801^1^ |
| **Disease duration** | 11.3 (2-21) | 6.7 (0-18) | n/a | n/a | 0.004^1^ |
| **Sex (% female)** | 7 (50%) | 13 (37.1%) | 6 (54.5%) | 15 (55.6%) | 0.8127 |
| **UPDRS-III mean, (SD)** | 21.5 (6.6) | 19.4 (11.4) | 1.0 (1.1) | 1.2 (1.5) | 0.536^1^ |
| **MoCA mean, (SD)** | 26.6 (2.5) | 26.5 (2.8) | 28.5 (1.0) | 27.3 (1.9) | 0.089 |
| **LEDD** | 661 (385) | 449 (403) | n/a | n/a | 0.099^1^ |
| **LRRK2 Activity Protocol A** | 59.7 (51.1) | 77.0 (46.4) | 93.1 (46.0) | 68.1 (34.4) | 0.367^2^ |
| **LRRK2 Activity Protocol B** | 110.2 (56.5) | 79.0 (65.4) | 144.9 (77.4) | 68.4 (42.8) | 0.012^3^ |
| **LRRK2 Activity Protocol C** | 70.3 (43.8) | 80.2 (76.4) | 69.8 (45.3) | 68.7 (49.7) | 0.900^4^ |

^1^The p-values were calculated to compare the two Parkinson’s disease groups with each other.

^2^ Includes 11 PD+*LRRK2*+, 28 PD+*LRRK2-*, 8 PD-*LRRK2*+, 22 PD-*LRRK2*-

^3^ Includes 11 PD+*LRRK2*+, 29 PD+*LRRK*2-, 8 PD-*LRRK2*+, 21 PD-*LRRK2*-

^4^ Includes 13 PD+*LRRK2*+, 29 PD+*LRRK*2-, 9 PD-*LRRK2*+, 25 PD-*LRRK2*-

**Table 2**. Antibodies used in the ELISA-based assays of LRRK2 function.

| **Antibody** | **Epitope** | **Company/Clone** | **Use** |
| --- | --- | --- | --- |
| LRRK2 | LRR | Abcam; c41-2 | Capture |
| LRRK2 | C-term | NeuroMab; N241A | Detector; total LRRK2 |
| LRRK2 | 1-500 aa | Abcam; UDD3 | Detector; total LRRK2 |
| pS935-LRRK2 | Phospho-Ser935 | Abcam; UDD2 | Detector; phosphorylated LRRK2 |
| Anti-pThr x Arg/pERM | Phospho Thr | Cell Signaling Technologies | Detection of phosphorylated peptide substrates |
